# Supplementary material for: Adjusting the Structure of β-Cyclodextrin to Improve Complexation of Anthraquinone-Derived Drugs
Source: Molecules. 2021 Nov 27;26(23):7205. doi: 10.3390/molecules26237205 (PMC8659250; doi:10.3390/molecules26237205)
Supplement: Supplementary file 1 [file molecules-26-07205-s001.zip › molecules-1407194-supplementary.pdf]

Supplementary Materials

Adjusting the Structure of β-Cyclodextrin to Improve Complexation of Anthraquinone-Derived Drugs

Agata Krzak[1,2], Olga Swiech[1,2*], Maciej Majdecki[3], Piotr Garbacz[1], Paulina Gwardys[1], Renata Bilewicz[1,2*]

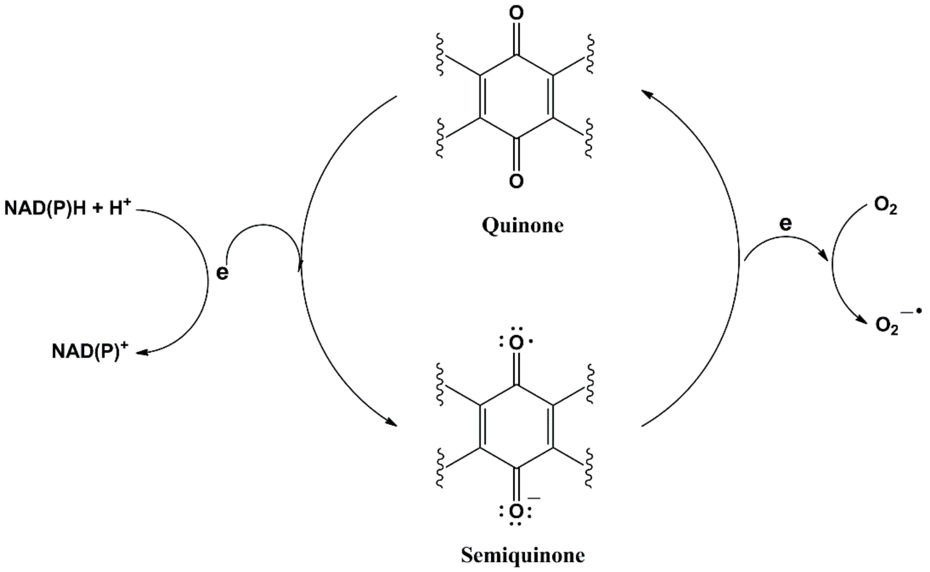

Scheme S1. Reactive oxygen species (ROS) production in the presence of NADH.

NMR measurements

The acquired NMR spectra of *β*-CD complexes are listed in Table S1. ^{1}H NMR signals of aromatic protons of the ligands, *i.e.*, AQ2S and AQ2CA (Figs. S4 and S8), almost do not overlap with the ^{1}H NMR signals of *β*-CDGAL (Fig. 1). Therefore, the ^{1}H NMR signals of aromatic protons are the most favorable for analyzing the complexes' structures (Figs. S6 and S10). In the case of DNR, there is a partial overlapping of the NOESY cross-peaks of DNR protons and those due to the interaction between *β*-CDGAL and DNR (see Figs S12−15). This overlapping hinders the unequivocal assignment of the signals. ^{1}H NMR of AQ2CA was recorded in DMSO-d_6 due to its very low solubility in water.

Table S1. NMR spectra of *β*-CD complexes given in the supporting material.

Spectrum	Measurement	Sample composition	
S1	^{1}H		
S2	^{1}H NOESY	β-CDGAL	
S3			
S4	^{1}H		AQ2S
S5	^{1}H		
S6	^{1}H NOESY	β-CDGAL	AQ2S
S7			
S8	^{1}H		AQ2CA
S9	^{1}H		
S10	^{1}H NOESY	β-CDGAL	AQ2CA
S11			
S12	^{1}H		DNR
S13	^{1}H NOESY		
S14	^{1}H	β-CDGAL	DNR
S15	^{1}H NOESY		

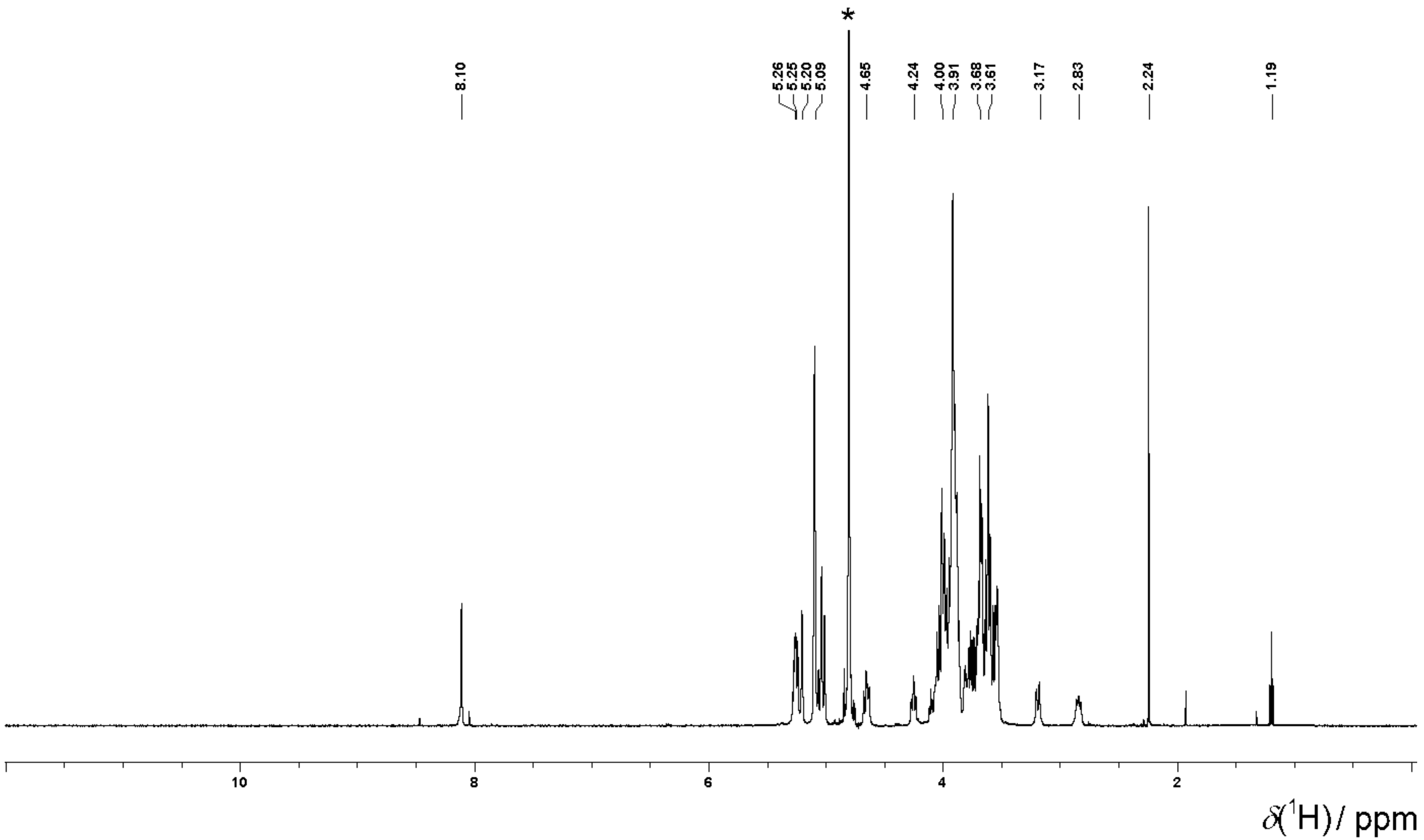

Figure S1. ^{1}H NMR spectrum of β-cyclodextrin functionalized by galactosamine (β-CDGAL) dissolved in D_2O. The residual solvent peak at $\delta(^{1}H) = 4.79$ ppm is marked by an asterisk.

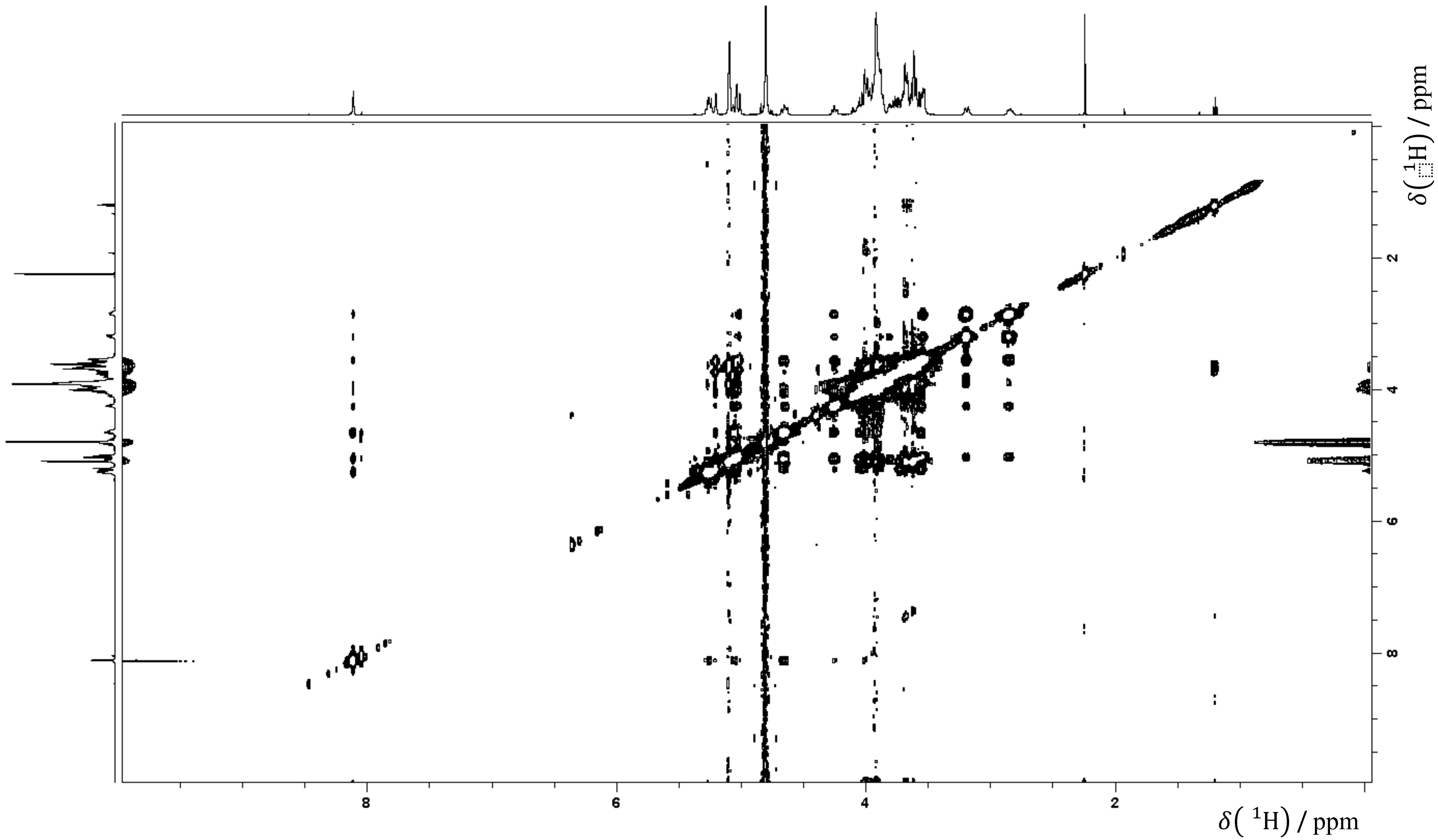

Figure S2. ^{1}H NOESY spectrum of *β*-CDGAL dissolved in D_2O.

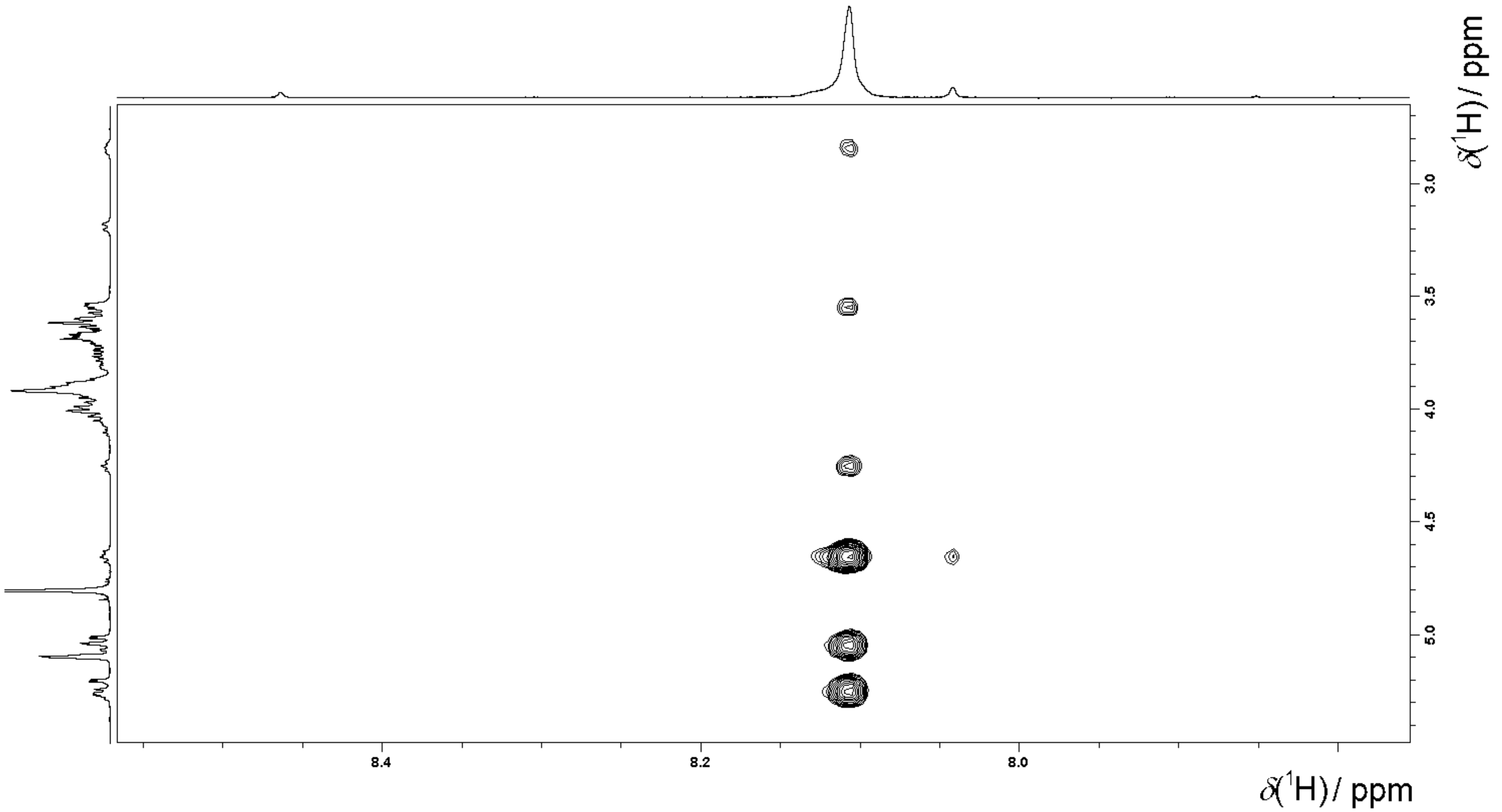

Figure S3. The spectral region of the 1H NOESY spectrum given in Fig. S2 showing the interaction between AQ2S and *β*-CDGAL.

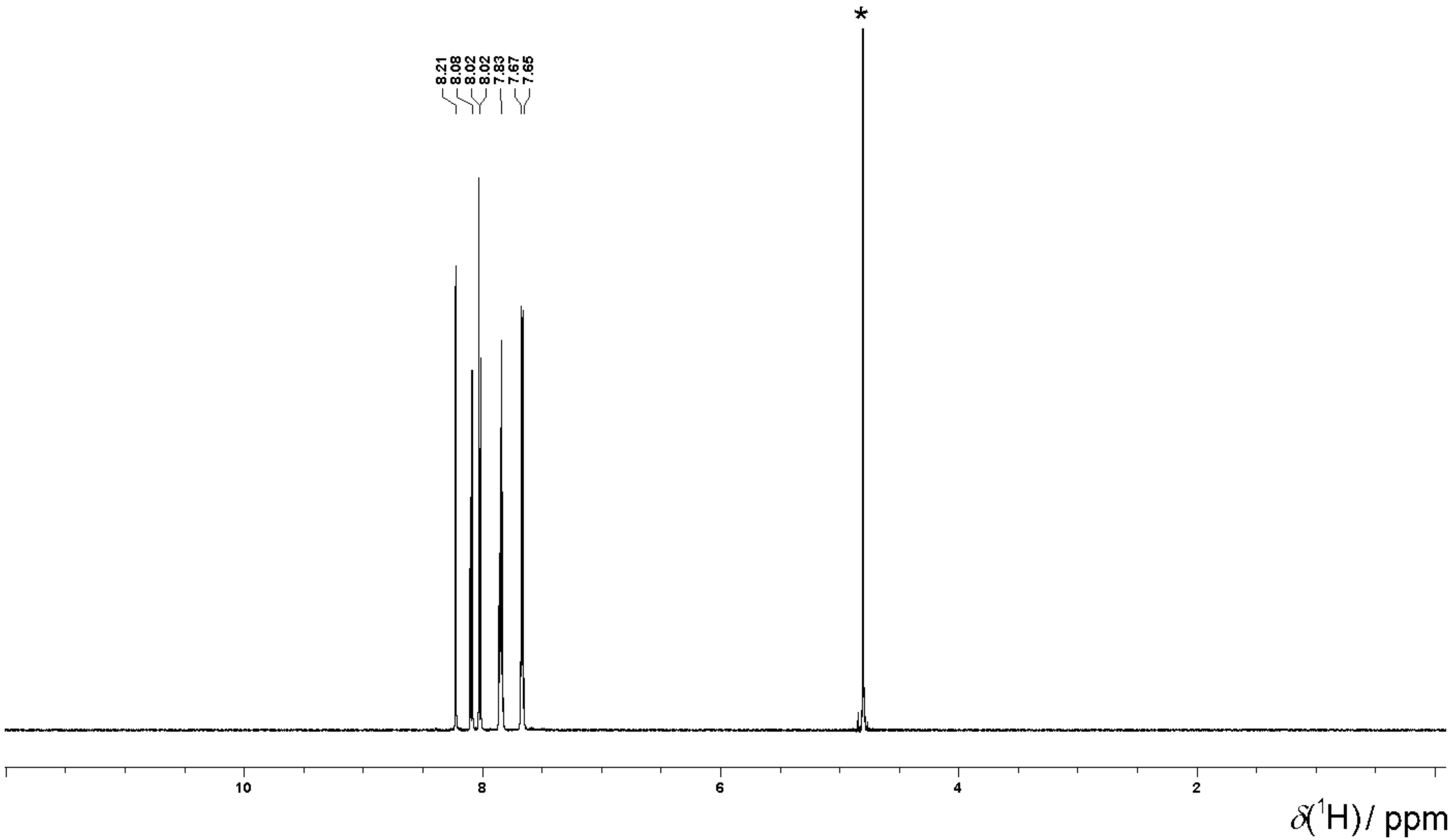

Figure S4. ^{1}H NMR spectrum of antraquinone-2-sulfonic acid (AQ2S) dissolved in D_2O. The residual solvent peak at $\delta(^{1}\mathrm{H}) = 4.79$ ppm is marked by an asterisk.

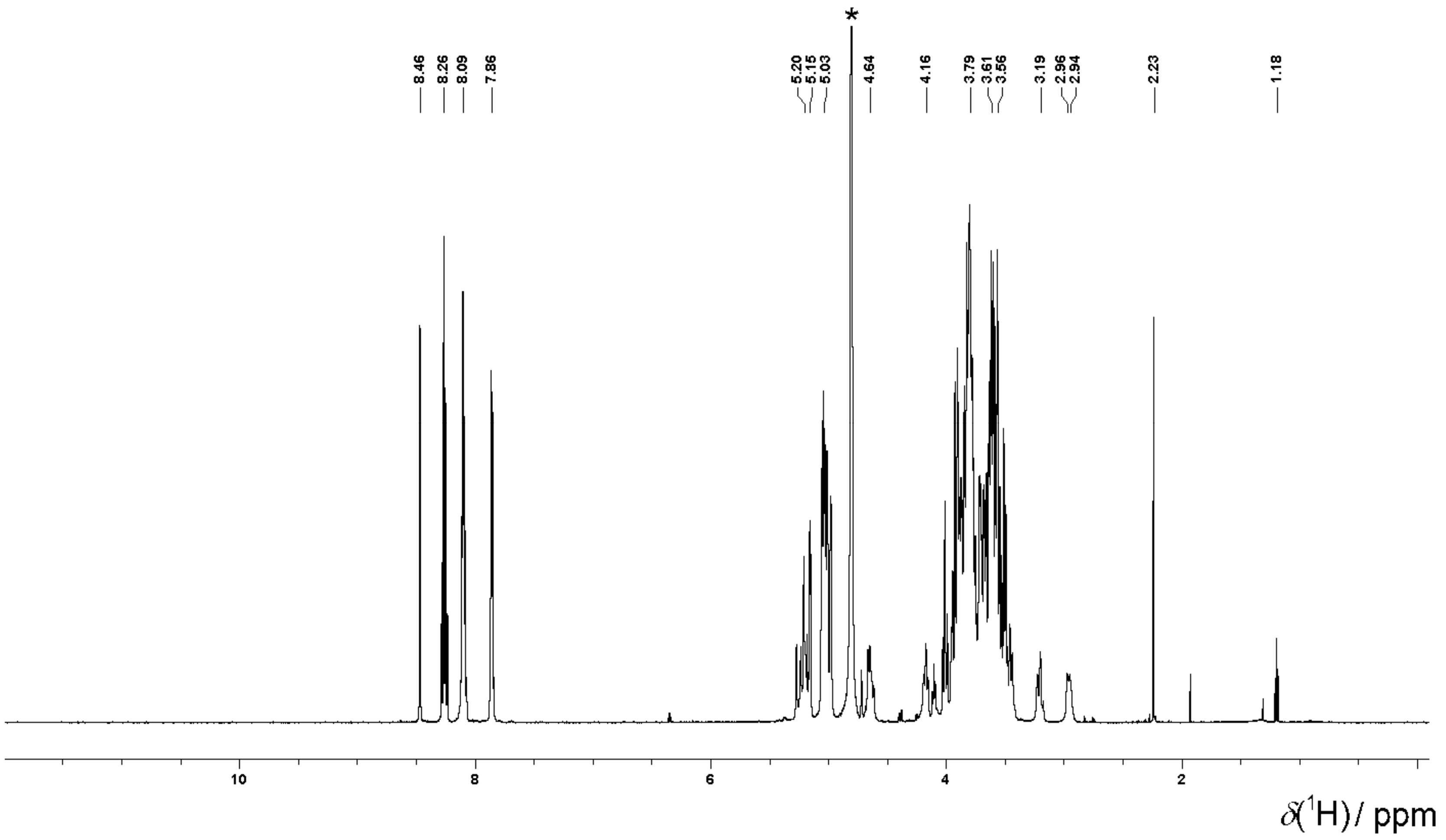

Figure S5. ^{1}H NMR spectrum of an equimolar mixtrure of AQ2S and *β*-CDGAL dissolved in D_2O. The residual solvent peak at $\delta(^{1}\mathrm{H}) =$ 4.79 ppm is marked by an asterisk.

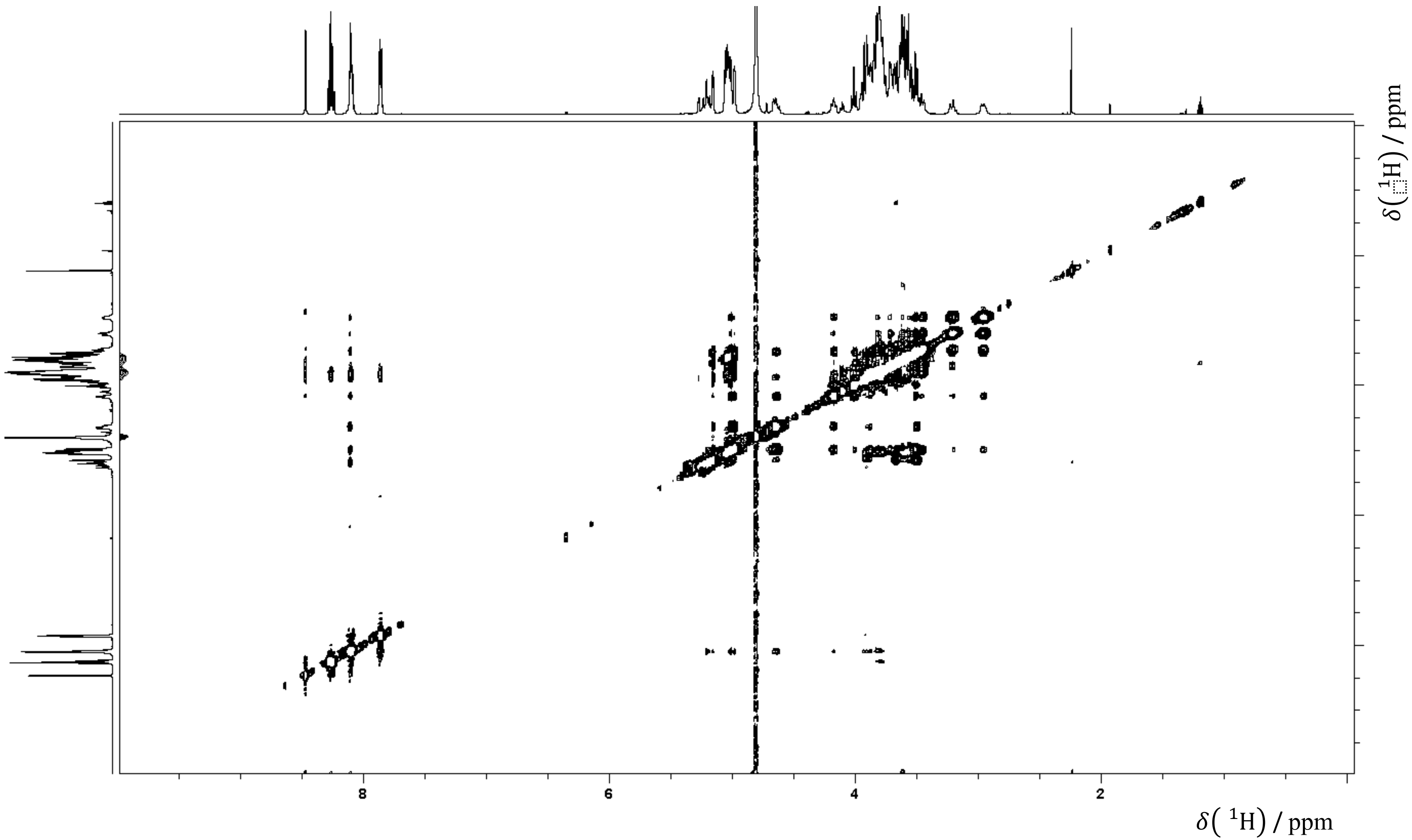

Figure S6. ¹H NOESY spectrum of an equimolar mixtrure of AQ2S and *β*-CDGAL dissolved in D_2O.

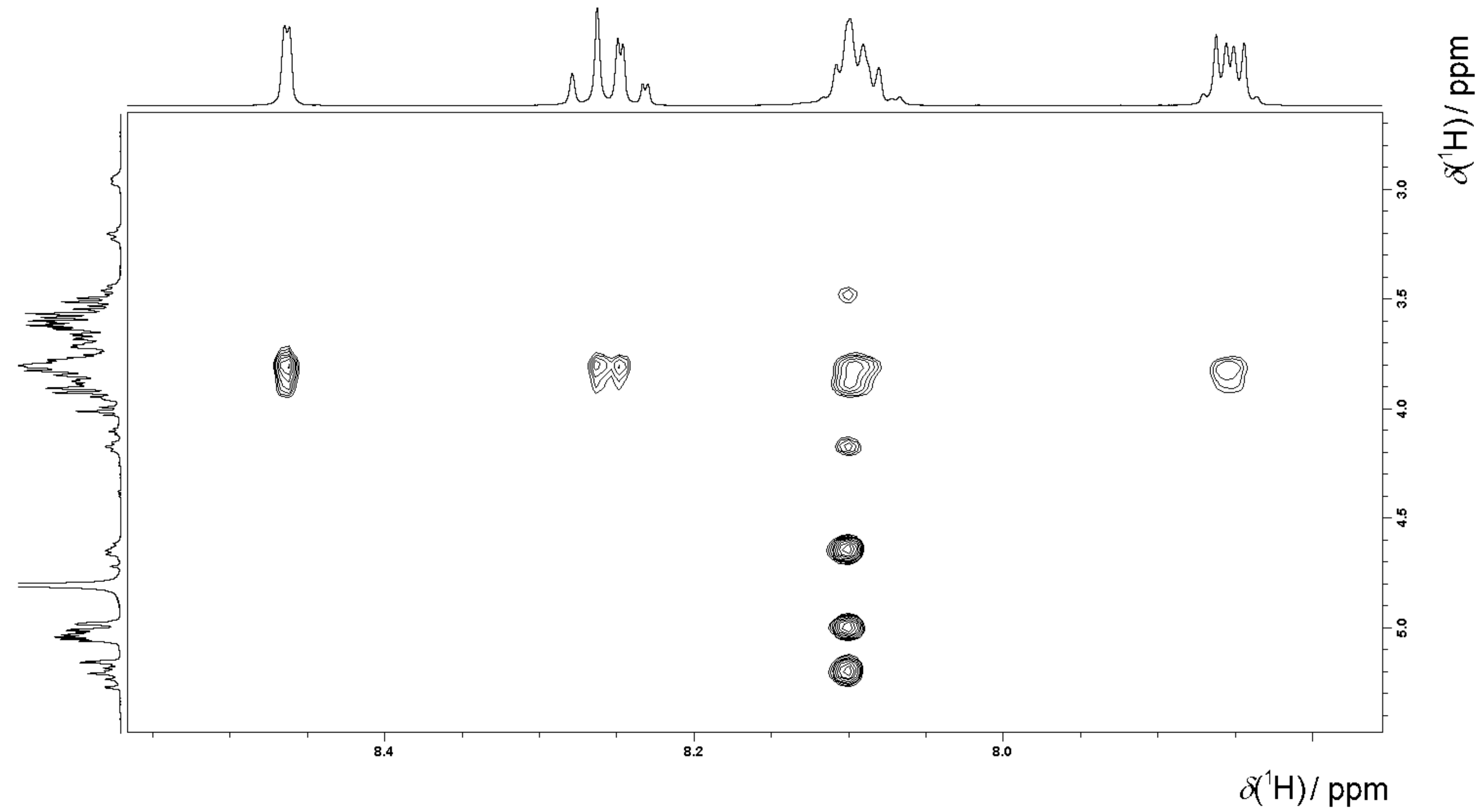

Figure S7. The spectral region of the ^{1}H NOESY spectrum given in Fig. S6 showing the interaction between AQ2S and *β*-CDGAL.

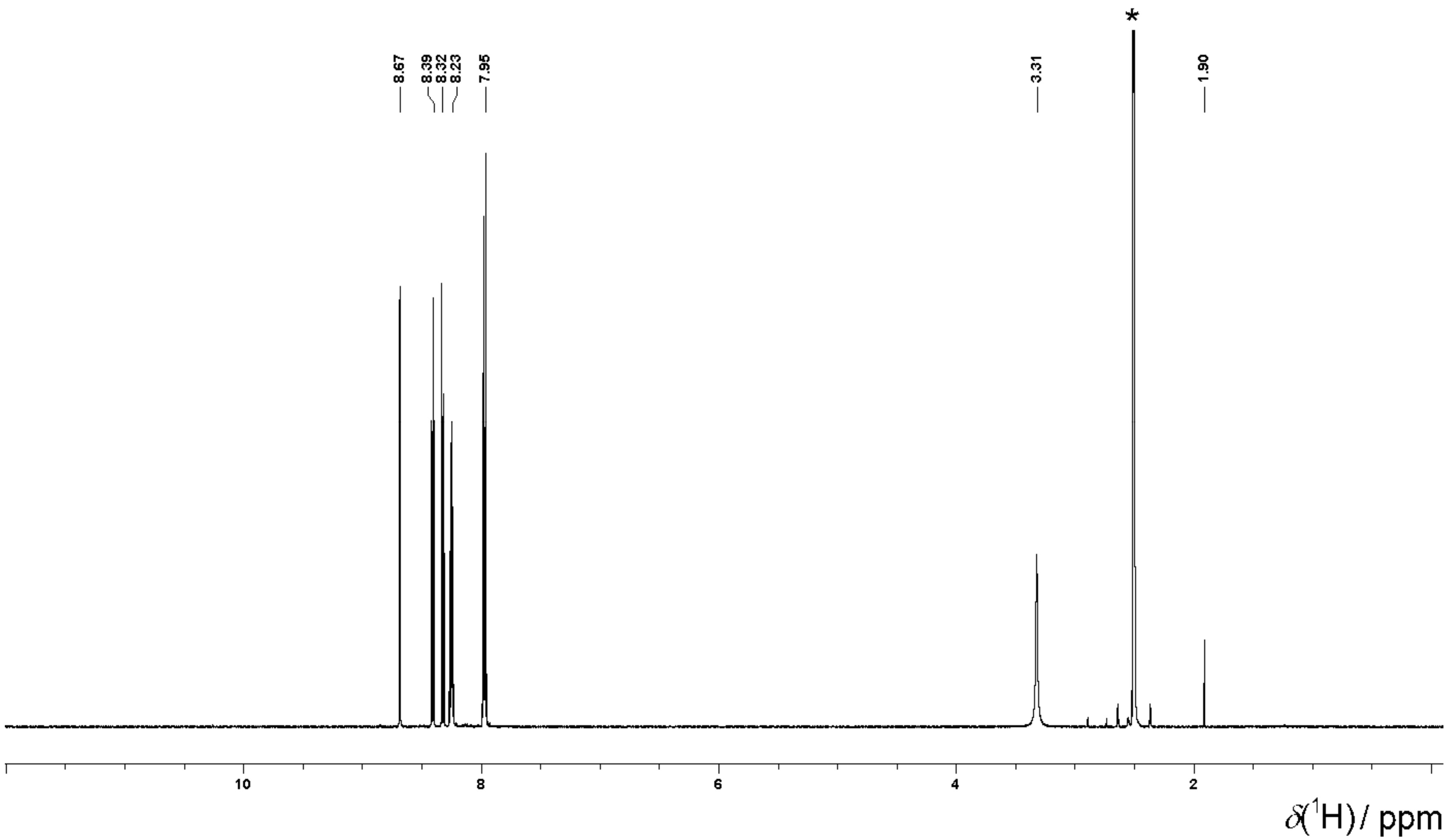

Figure S8. ^{1}H NMR spectrum of antraquinone-2-carboxylic acid (AQ2CA) dissolved in DMSO-d_6. The residual solvent peak at $\delta(^{1}\mathrm{H}) = 2.50$ ppm is marked by an asterisk.

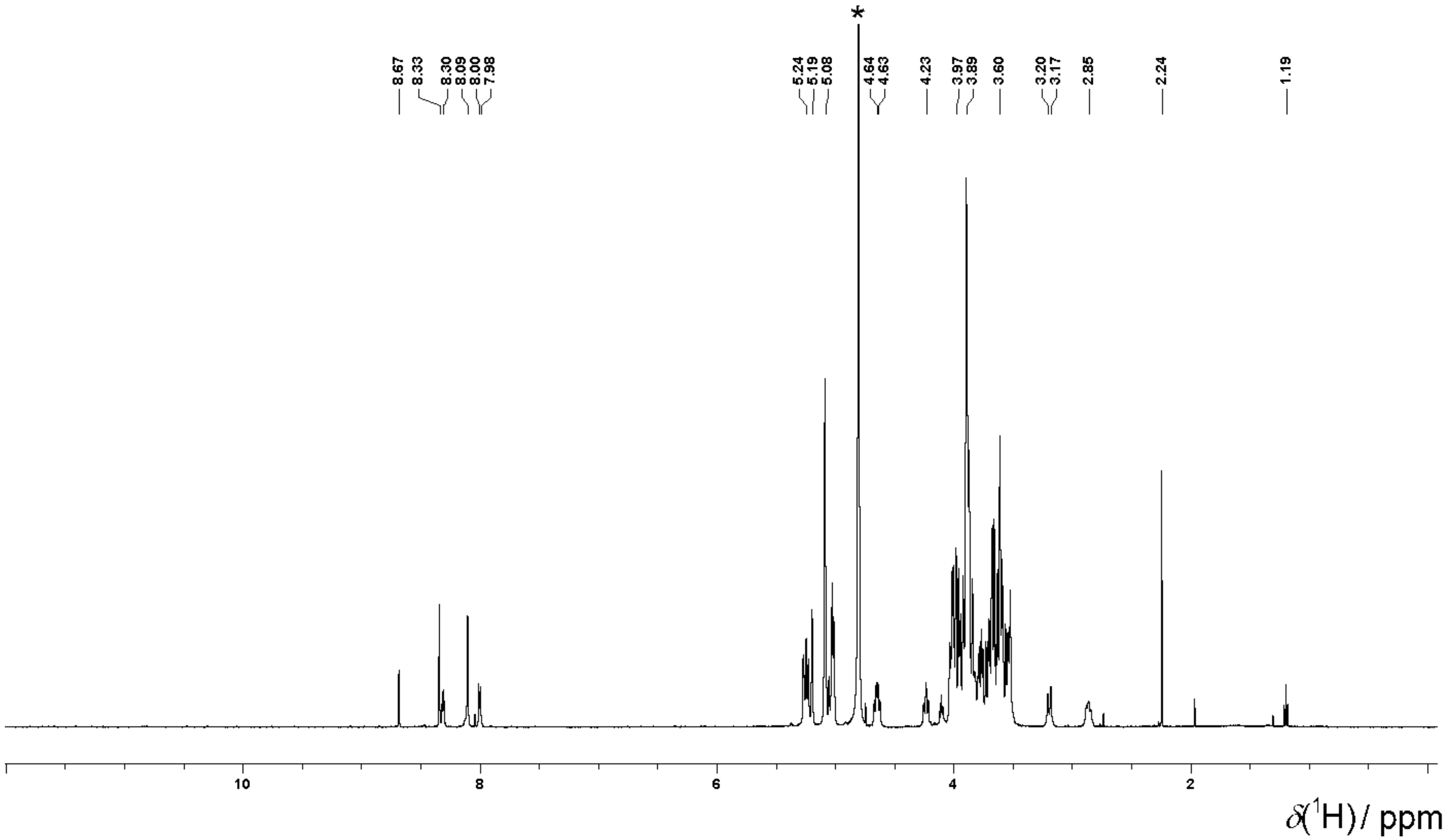

Figure S9. ^{1}H NMR spectrum of an equimolar mixtrure of AQ2CA and β-CDGAL dissolved in D_2O. The residual solvent peak at $\delta(^{1}\mathrm{H}) = 4.79$ ppm is marked by an asterisk.

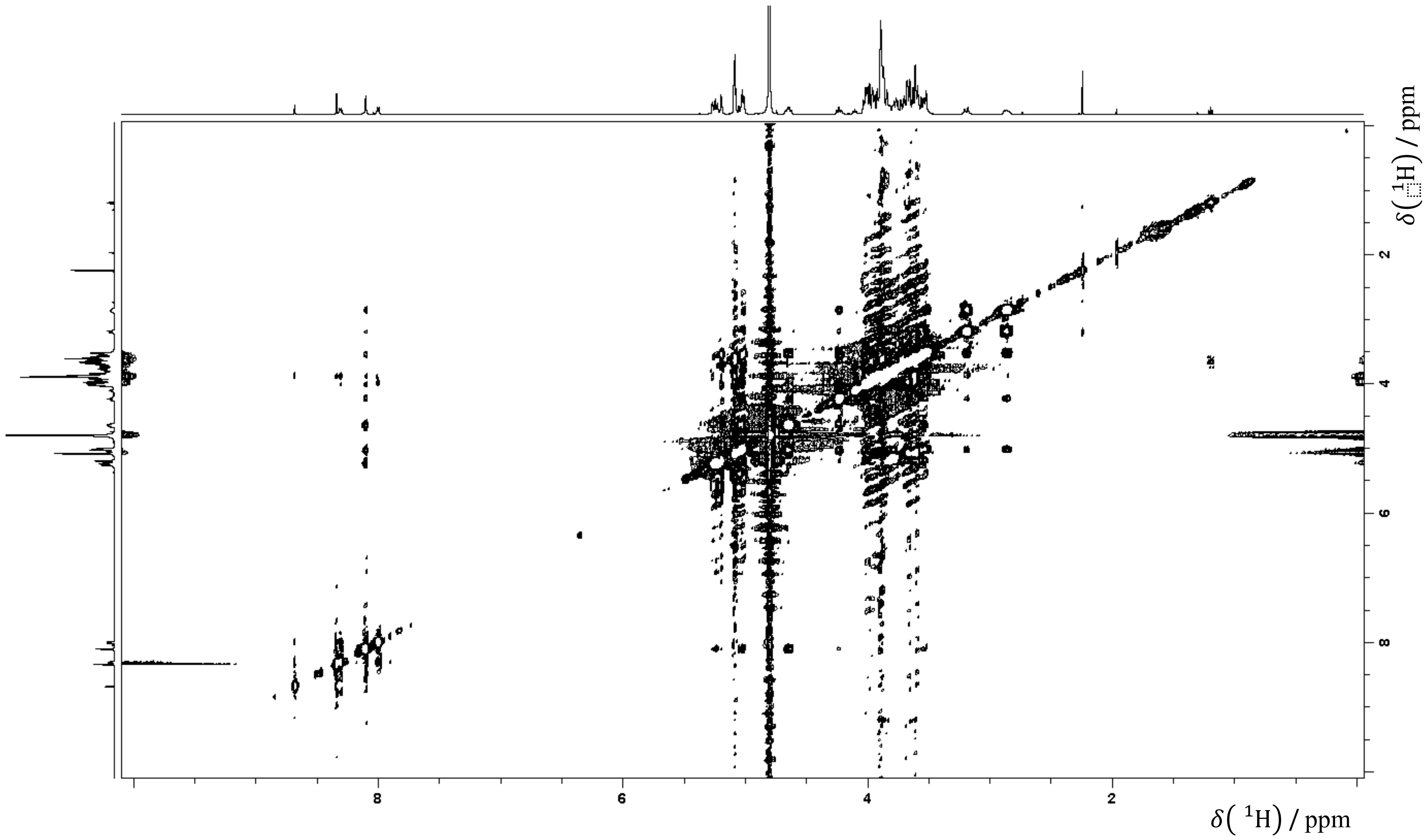

Figure S10. ^{1}H NOESY spectrum of an equimolar mixtrure of AQ2CA and *β*-CDGAL dissolved in D_2O.

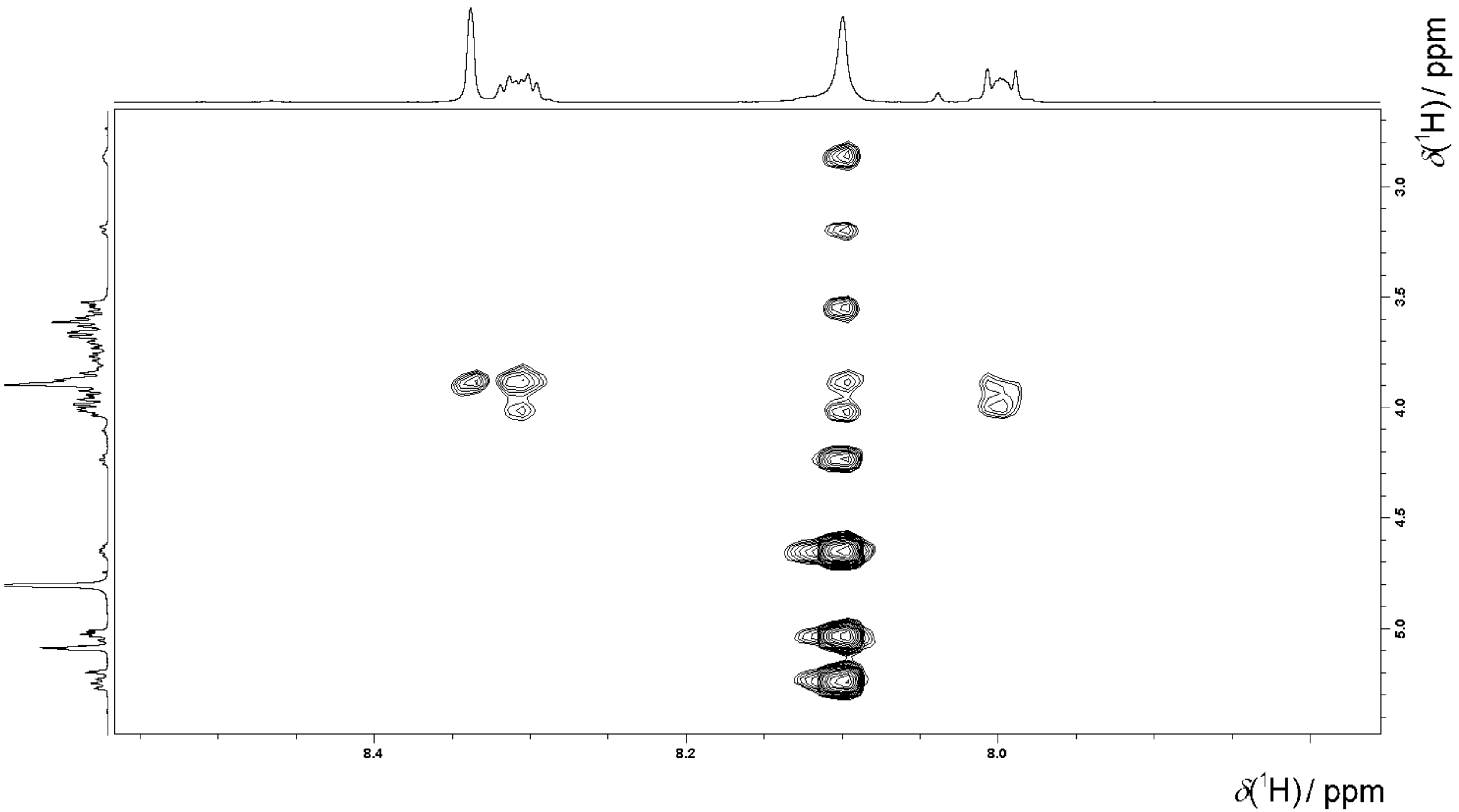

Figure S11. The spectral region of the ^{1}H NOESY spectrum given in Fig. S10 showing the interaction between AQ2S and *β*-CDGAL.

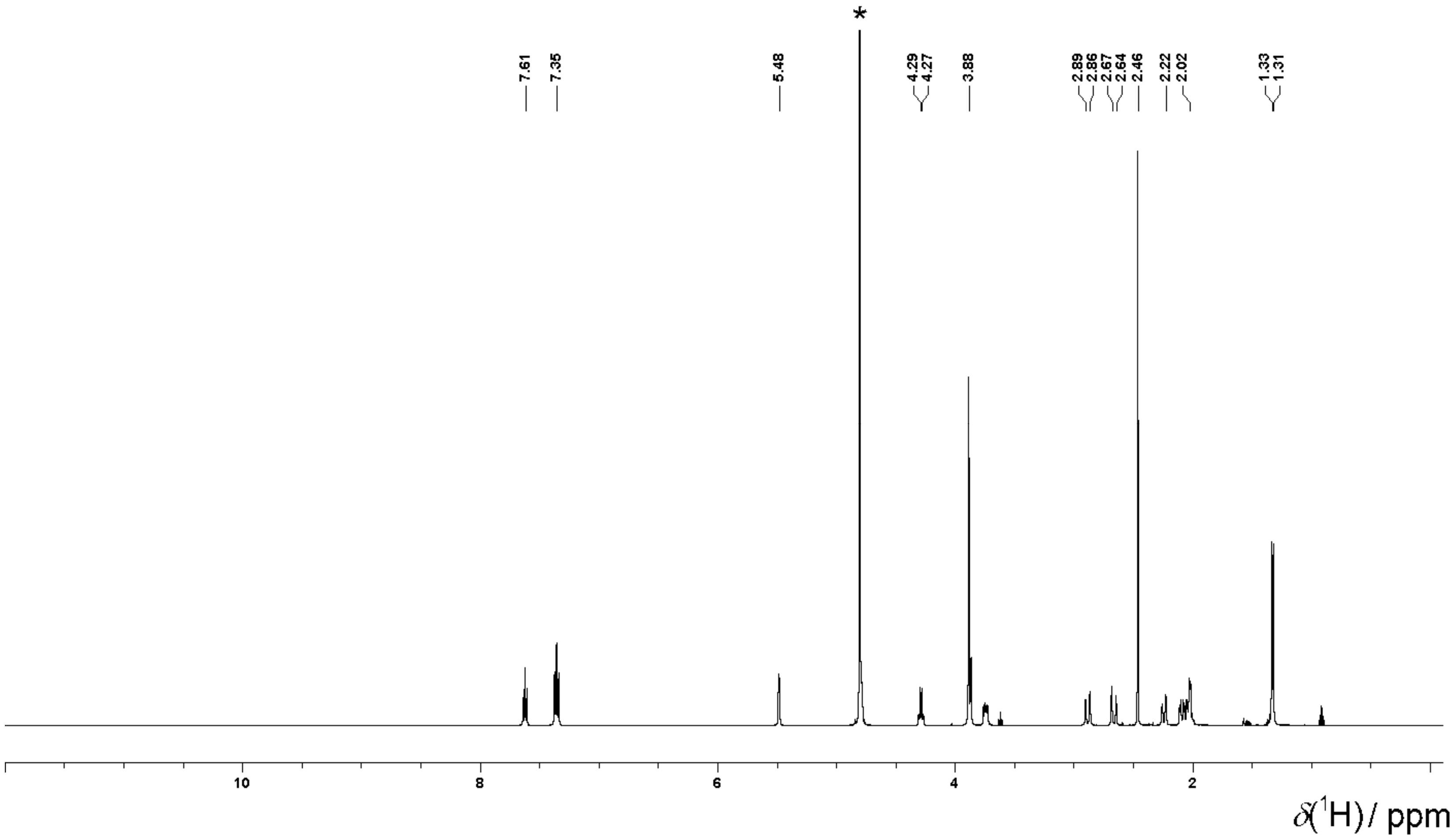

Figure S12. ^{1}H NMR spectrum of daunorubicin (DNR) dissolved in D_2O. The residual solvent peak at $\delta(^{1}H) = 4.79$ ppm is marked by an asterisk.

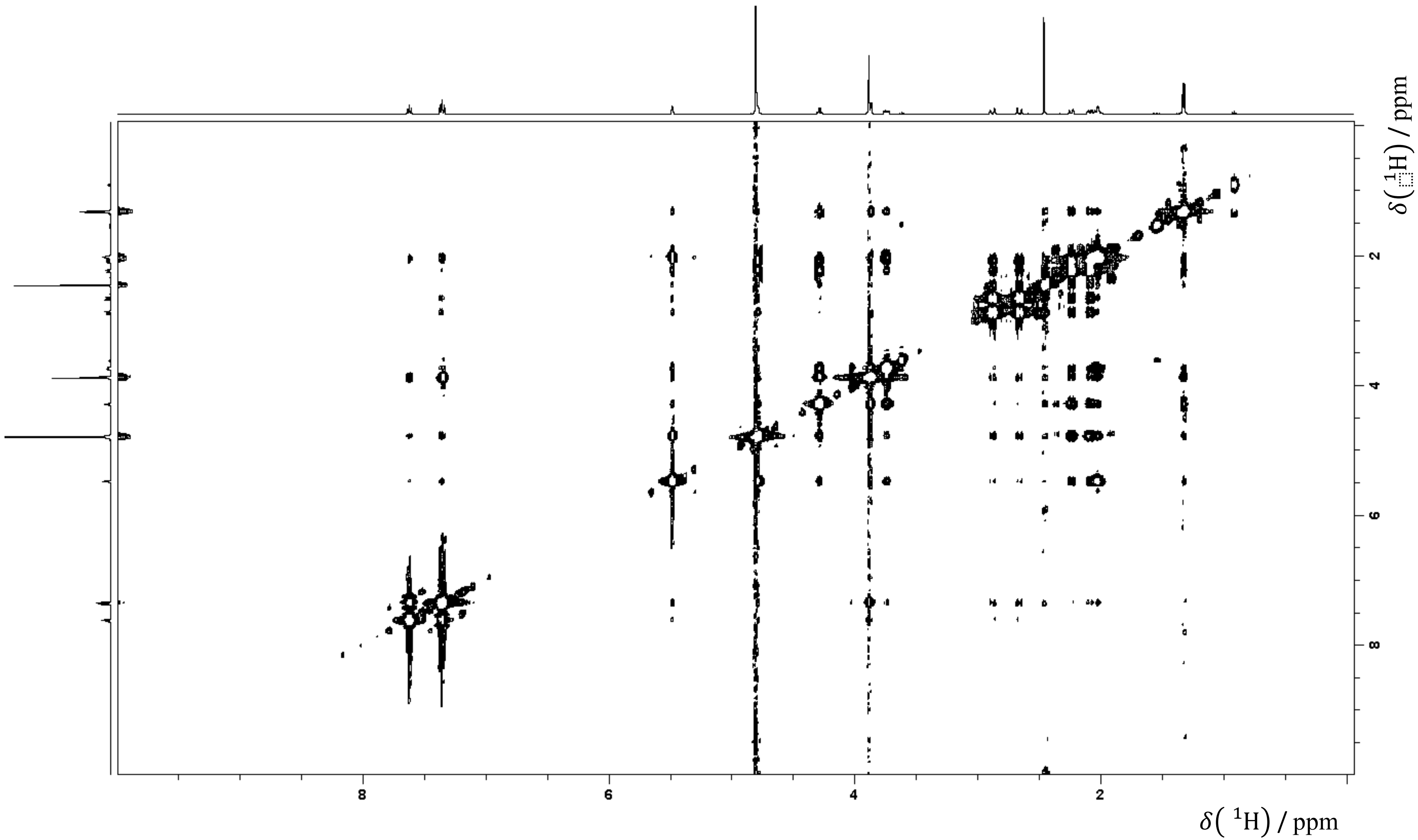

Figure S13. ^{1}H NOESY spectrum of DNR dissolved in D_2O.

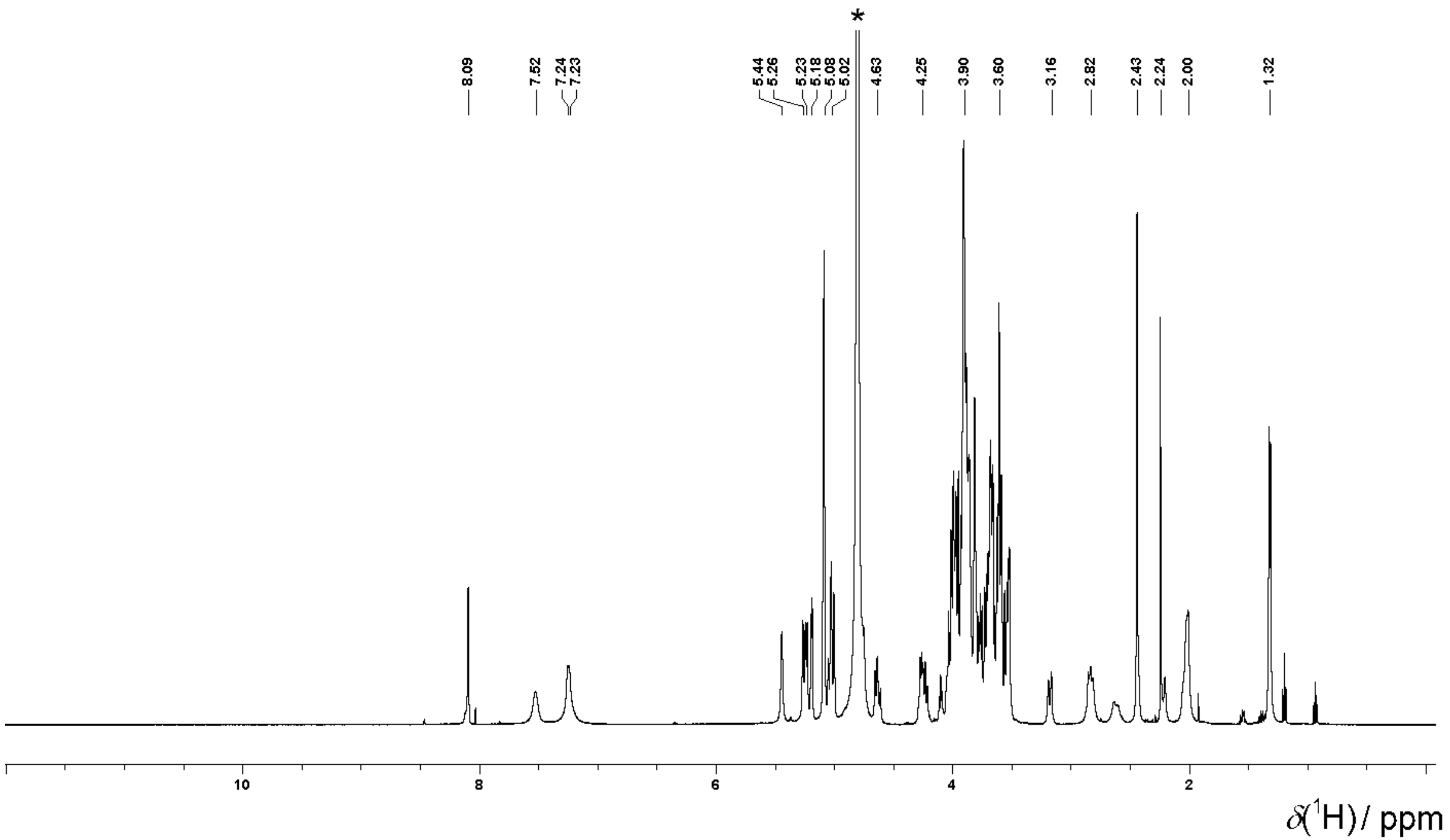

Figure S14. ^{1}H NMR spectrum of an equimolar mixtrure of DNR and *β*-CDGAL dissolved in D_2O. The residual solvent peak at $\delta(^1\mathrm{H}) = 4.79$ ppm is marked by an asterisk.

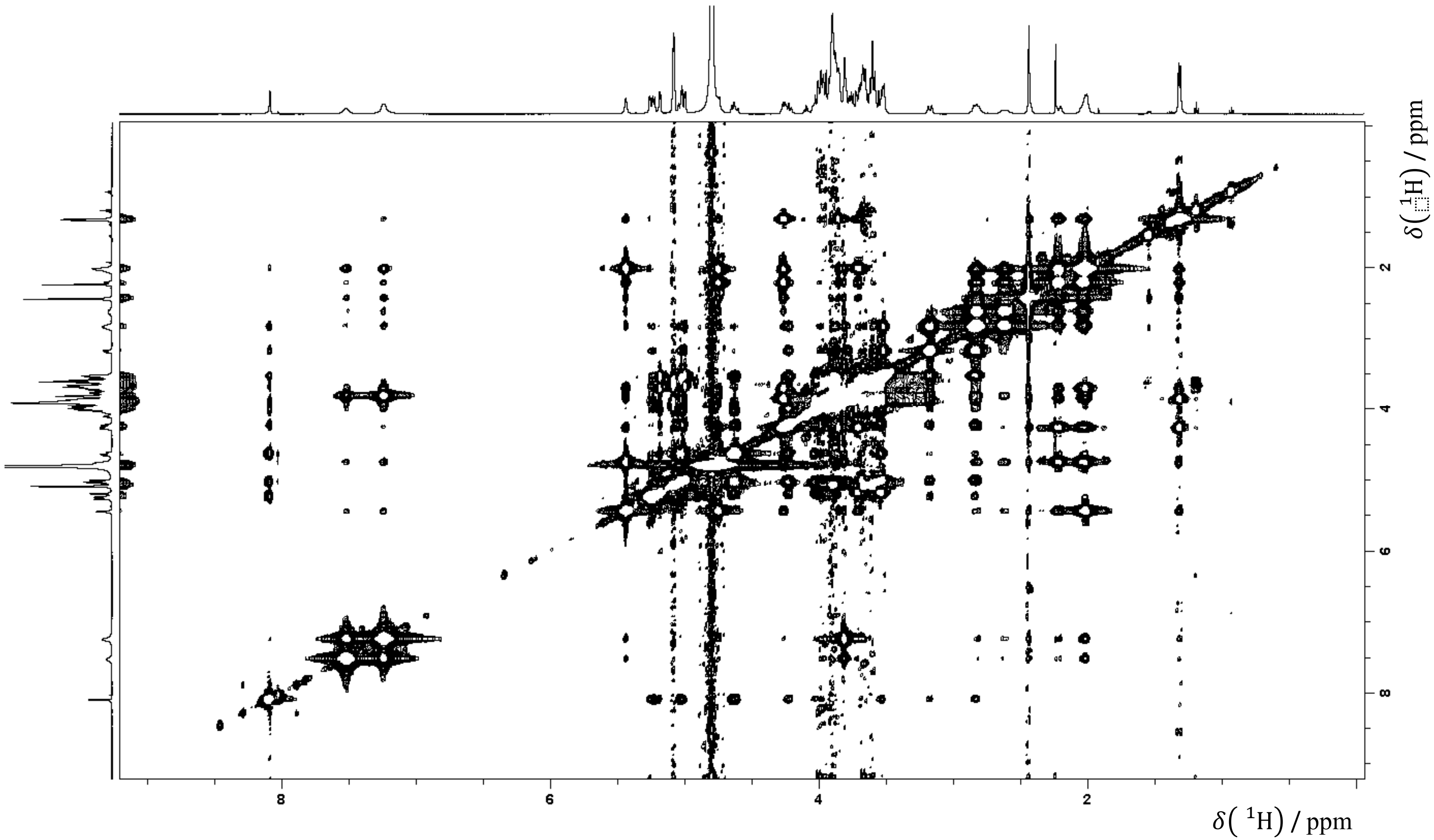

Figure S15. ^{1}H NOESY spectrum of an equimolar mixtrure of DNR and *β*-CDGAL dissolved in D_2O.
